# Orthodontic treatment time: can it be shortened?

**DOI:** 10.1590/2177-6709.23.6.090-105.sar

**Published:** 2018

**Authors:** Ricardo Moresca

**Affiliations:** 1 Universidade Federal do Paraná, Programa de Pós-graduação em Ortodontia (Curitiba/PR, Brazil).

**Keywords:** Orthodontics, Corrective orthodontics, Tooth movement techniques.

## Abstract

**Introduction:**

In the literature, no consensus has been reached about orthodontic treatment time. Similarly, the determining factors of the latter have not yet been completely elucidated.

**Objective:**

The aim of the present article was to deepen the discussion on the major factors influencing orthodontic treatment time, as well as to present some strategies that have proven effective in controlling and shortening it.

**Method:**

Based on evidences found in the literature, the method focussed in providing the basis for clinical decision-making.

**Conclusions:**

Treatment time varies according to the type of malocclusion and treatment options. Orthodontist’s influence, patient’s characteristics and compliance are all decisive in determining treatment time, while the effects provided by orthodontic appliances and methods used to speed tooth movement up seem little effective.

## Resumo

**Introdução:**

não existe consenso na literatura sobre a duração do tratamento ortodôntico, e os fatores relacionados com a sua determinação ainda não estão totalmente elucidados.

**Objetivo:**

o objetivo do presente artigo foi aprofundar a discussão dos principais fatores que podem influenciar na duração do tratamento ortodôntico, bem como apresentar algumas estratégias que têm se mostrado eficientes para controlá-la e reduzi-la.

**Método:**

a partir das evidências apresentadas na literatura, fundamentar as decisões clínicas.

**Conclusões:**

o tempo de tratamento varia de acordo com o tipo de má oclusão e com as opções de tratamento. A influência do ortodontista, as características do paciente e sua colaboração são fatores decisivos na definição do tempo de tratamento, enquanto os efeitos dos dispositivos ortodônticos e os métodos de aceleração do movimento dentário parecem ser pouco efetivos.

## INTRODUCTION

“How long will I be using braces?”. This is one of the questions patients ask the most in regard to orthodontic treatment. 

In fact, the question reveals a strong desire, especially by adult patients, for shorter treatment, since the anti-aesthetic look provided by orthodontic brackets in addition to longer correction time are the major factors responsible for demotivating patients to have treatment began.^1^

In the literature, no consensus has been reached about orthodontic treatment time. A recent systematic review revealed mean treatment time with fixed appliances of 19.9 months. However, there was significant variation among studies (with mean values ranging from 14 to 33 months), and the quality of treatment outcomes was not assessed.^2^ Whenever cases were assessed under the American Board of Orthodontics (ABO) standards, one-phase orthodontic treatment mean time was 24.6 months.^3^ ^,^ ^4^

In Brazil, studies assessing orthodontic treatment time suggest variation is within world average.^5^ ^-^ ^8^

On the other hand, orthodontic treatment mean time seems to be beyond patients’ expectation. When asked about how long they would like treatment to last, 40.8% of adolescent patients answered less than 6 months, while 33.2% of them answered between 6 and 12 months. Among adult patients, 42.9% answered between 6 and 12 months, while 26.5% answered between 12 and 18 months.^9^

Extremely long treatment time has been associated with greater susceptibility to iatrogenesis, which in turn are associated with orthodontic appliances. This is the case of root resorption, white spots, carious lesions, and gingival inflammation.^10^

Furthermore, patients’ quality of life and self-esteem can be harmed as a result of fixed appliances use, as they may lead to discomfort and trouble relative to their daily routine. Additionally, fixed appliances add extra appointments to patients’ agenda. The aforementioned factors are probably associated with the fact that longer-than-expected treatment time is one of the major causes of patient dissatisfaction.^1^

In contrast to what could have been expected, longer treatment has been associated with worse or unacceptable occlusal outcomes.^4^ Such an association might be related to primary factors, such as mistaken diagnosis and planning, as well as lack of patient compliance. A more accurate estimate for orthodontic treatment time can help giving a more realistic estimate of treatment costs, in addition to minimizing risks of iatrogenesis, as well as increasing success rates and patient’s satisfaction. Thus, being aware of the factors influencing orthodontic treatment time and determining efficient control mechanisms are worthwhile.

Based on information found in the literature as well as on clinical investigation, the aim of the present study was to deepen discussion on the major factors influencing orthodontic treatment time, as well as to present some strategies that have proven efficient at both controlling and shortening it.

## TYPES OF MALOCCLUSION AND TREATMENT OPTIONS

Orthodontic treatment time can be influenced by malocclusion characteristics and treatment methods. 

### Malocclusion severity

More complex cases tend to take longer to be corrected.^3^ ^,^ ^11^ ^,^ ^12^ The ABO Discrepancy Index (DI) has shown a positive association with orthodontic treatment time. Cases with DI > 15 were significantly longer (30 months) than cases with DI ≤ 15 (22 months). Should DI be greater than 15, treatment time is expected to last more than 22.1 months in 85% of cases.^3^

### Premolar extractions

Despite controversy,^13-15^ premolar extractions tend to increase treatment time.^4^ ^,^ ^5^ ^,^ ^8^ ^,^ ^12^ ^,^ ^14^ ^,^ ^16^ ^,^ ^17^ Such an increase can be explained by an association between extractions and more complex cases, as well as the need of an additional treatment step aimed at space closure.^4^

In borderline cases, using interproximal stripping to avoid extractions may shorten treatment time in eight months.^17^

With first premolar extractions, treatment time was similar for both Class I and Class II (28.95 months and 28.10 months, respectively); however, with better occlusal outcomes in Class I.^7^

The number of extractions also influences treatment time. For cases without extraction, with two extractions, and with four extractions, mean treatment time was 21.95 months, 25 months, and 26.18 months, respectively.^18^

Nevertheless, the literature is inconclusive when determining additional time necessary for extraction cases, ranging from 1.4 month^14^ to 7.8 months.^11^ Such variation might be related to the amount of space to be closed and tooth movement rate. Factors such as age, which teeth will be extracted, number of extractions,^5,18^ degree of crowding,^8^ mechanics of choice,^19^ and planning^7,17^ (degree of anterior retraction and anchorage level) must be assessed.

In regard to mass retraction of anterior teeth carried out with sliding mechanics, monthly space closure rate was 0.35 mm for steel ligatures associated with elastomeric modules having 3-mm activation, 0.58 mm for elastomeric chains (doubly stretched in comparison to original size), and 0.81 mm for NiTi springs (200 gf).^19^

Once extractions of maxillary teeth were the only ones assessed in adolescent patients using sliding mechanics and NiTi springs (150 gf), monthly space closure rate was 1.22 mm for the left and 1.35 mm for the right side.^20^

### Class II

Class II treatment lasts on average from 5 to 7.4 months more than Class I treatment.^3^ ^,^ ^11^ ^,^ ^15^ ^,^ ^16^ In addition to molar relationship, ANB angle,^15^ ^,^ ^18^ overjet equal or greater than 5 mm^12,15^ and vertical pattern^18^ can also contribute to longer Class II treatment. 

Correction methods also influence treatment time. Extraoral anchorage can increase treatment time in six months.^11^ ^,^ ^14^ Using the Herbst appliance led to increased treatment time in 8 to 9 months,^11,15^ while rapid maxillary expansion added 3.4 months to treatment time.^11^

Elastics have also been associated with increased Class II treatment time.^15^ In comparison to elastics, the use of Forsus decreased treatment time in 2.5 months.^21^

Two-phase Class II treatment has not proven more efficient and tends to last longer than one-phase treatment^13,14^ (up to eight months more).^14^

Most studies reveal Class II treatment associated with extractions lasts longer.^5^ ^,^ ^8^ ^,^ ^18^ Class II treatment protocol encompassing extractions of two maxillary teeth not only results in better occlusal outcomes than the four-extraction protocol, but also shortens treatment time.^5^ ^,^ ^7^ Class II treatment associated with four extractions requires more complex mechanics as well as more patient compliance.^7^

Class II treatment associated with two extractions lasts on average 23.52 months, whereas treatment associated with four extractions lasts on average 28.12 months. In cases with no crowding but with more anterior retraction, mean treatment time changed to 24.35 and 30.13 months, respectively.^5^ In cases with crowding, spaces are minimized at treatment onset, thus decreasing the amount of movement and shortening the time required for space closure.

### Class III

Despite little information available on Class III treatment time, the non-surgical approach seems to last longer (30.27 months) than treatment of other sagittal malocclusions.^11^ Treatment of patients with SNB < 76° has two to three more chances to last longer than 30 months.^12^

Due to treatment methods, Class III treatment time seems to be strongly influenced by factors relative to patient compliance.

### Orthognathic surgery

Orthodontic treatment associated with orthognathic surgery may result in longer treatment time, despite the wide variation previously reported. On average, this treatment modality may last from 18 to 36 months, depending on skeletal disharmony and malocclusion severity, as well as on the type of surgery.^22^ ^-^ ^24^

The pre-surgical phase lasts on average 15 to 24 months, while the post-surgical one lasts from 6 to 12 months.^23^ ^,^ ^24^ When treatment was associated with extractions, pre-surgical phase time increased in 10 months on average.^24^

Treatment time may increase as a result of the need for transverse correction. This is because it is usually associated with more complex cases, longer time required for stabilization, and a more significant tendency towards relapse.^22^

## THE ORTHODONTIST’S INFLUENCE

Orthodontists play a key role in orthodontic treatment time, particularly considering their education and experience, treatment planning, standards of care, and the level of quality required for finishing. The aforementioned factors help to explain variations in treatment time found in different dental offices.^14^ Orthodontic treatment time is usually shorter when performed by more experienced clinicians.^13^

Diagnosis and planning mistakes, which lead not only to changes to treatment planning during correction, but also to late decision-making, hinder initial treatment time estimate.^15^

Contrary to what many techniques currently advocate, shorter intervals between appointments seem to contribute to keep treatment under control and result in shorter treatment time.^11^ ^,^ ^15^ ^,^ ^25^

The clinician’s demand for high quality and the time spent with finishing details also help to determine orthodontic treatment outcomes and time.^2^ ^,^ ^3^ ^,^ ^25^

Some clinicians have financial support as a result of delivering treatment with more efficient outcomes, which is associated with less and shorter appointments and greater patient satisfaction. On the other hand, many orthodontists fear that shorter treatment time will, in turn, decrease their financial income.^9^

## PATIENT’S TRAITS AND COMPLIANCE

Influence exerted by age, sex and socioeconomic level over orthodontic treatment time is not yet fully acknowledged.^3^ ^,^ ^6^ ^,^ ^12^ ^,^ ^16^

Despite age not being significantly associated with treatment time,^14,18^ it has been reported that the older the patient, the shorter treatment time will be, due to more significant compliance of older patients.^13^ ^,^ ^15^

Dental development stage is more decisive for treatment time than age. Presence of deciduous teeth at treatment onset is an indication of longer treatment.^12^

Patient-compliance-related factors, such as not attending to appointments, not using accessory devices, inefficient oral hygiene, and appliance breakage, are decisive to longer treatment.^11^ ^,^ ^14^ ^,^ ^16^ ^,^ ^18^ ^,^ ^26^

Each missed appointment, inefficient oral hygiene, unused elastics, and bracket or band replacement added 1 month, 0.67 month, 1.4 months and 0.6 month to treatment, respectively.^14^ ^,^ ^16^

It is interesting to note that patients’ motivation and compliance decrease as orthodontic treatment is delayed.^26^

Compliance is of paramount importance, therefore, it is suggested that patients’ motivation be kept throughout treatment. Texting patients via cellphone apps contributed to increase patient compliance, thus shortening treatment time in 7.3 weeks, resulting in 7% less missed appointments, 10% less late patients, and 4% less appliance breakage.^27^

## ORTHODONTIC APPLIANCES EFFECTS

The expectation that orthodontic appliances and the use of new technology will shorten treatment time has not been confirmed by most studies. 

No difference in orthodontic treatment time has been noticed relative to the type of brackets used: whether metallic or ceramic,^6^ ^,^ ^9^ ^,^ ^14^ conventional or self-ligating,^28^ or personalized ones.^29^

Although self-ligating brackets do not affect treatment time, they produce more effective sliding movement at initial correction. Nevertheless, the finishing phase might last longer, due to less effective rotational and torque control.^4^

Slot dimensions,^30^ prescription,^31^ and alignment wire sequence^32^ do not affect treatment time.

Temporary anchorage devices provide absolute anchorage and minimize undesired side effects; however, they do not shorten the time necessary for planned movements.^33^

Despite shortening appliance placement time and offering patients comfort, the assumption that indirect bonding can shorten treatment time has not yet been confirmed.^34^

Aligners shortened treatment time in 5.7 months (30%), in comparison to fixed appliances. However, fixed appliances were more effective than aligners in improving malocclusion. The likelihood of aligners improving malocclusion was 0.329 times the likelihood of fixed appliances achieving the same outcome.^3^ ^5^

## TOOTH MOVEMENT SPEEDING-UP METHODS

In the last few years, a number of different techniques have been suggested to speed up tooth movement. Nevertheless, evidence on efficiency of the majority of those methods is insufficient, in addition to their high costs and low acceptance by orthodontists and patients to more invasive surgical procedures.^2^ ^,^ ^9^

Tooth movement speeding-up methods can be grouped as mechanical or physical stimulation and facilitating surgical procedures. 

### Mechanical or physical stimulation

The quality of evidence proving that laser therapy can speed orthodontic movement up is low.^36^

The use of vibrational force has been proved not to affect tooth movement rates with fixed appliances^20^ or aligners.^37^

### Facilitating surgical procedures

Of the surgical procedures presently performed, corticotomy has presented some evidence of speeding orthodontic movement up. However, it has been characterized by a temporary and short speeding-up phase.^38^

Piezocision^39^ and micro-osteoperforations^40^ have not proven capable of changing tooth movement pattern.

## STRATEGIES TO CONTROL AND SHORTEN TREATMENT TIME

Based on diagnosis and treatment planning, a few measures can be adopted with a view to contributing to treatment time control or shortening.

### Defining the problem

An important measure to be taken in order to control treatment time is to precisely determine treatment goals. It is also recommended that the orthodontist ask patients about their expectations.

Given that most patients do not seek ideal occlusal outcomes, treatment goals and time can be adjusted to achieve specific results. This can be done on the basis of satisfactory occlusal stability.

Interaction between Orthodontics and other dental specialties can also help to improve treatment outcomes and shorten treatment time. 

#### Suggestion

» Include the following during patients’ first interview: their expectations, level of quality required, acceptance of orthodontic devices, motivation, and compliance profile.

### Treatment time individual estimate

One of the main reasons of patients’ dissatisfaction is noncompliance with treatment time initially proposed. 

Therefore, once treatment goals have been set, it is important to make a precise and individual estimate of the time required for the intended correction. 

A productive way to make a better estimate of treatment time is to divide it into phases. For each phase, a time estimate can be made, and a treatment step-by-step schedule determined. As a result, occasional diversion from what is initially proposed can be quickly identified.

#### Suggestions

**Figure 1:**
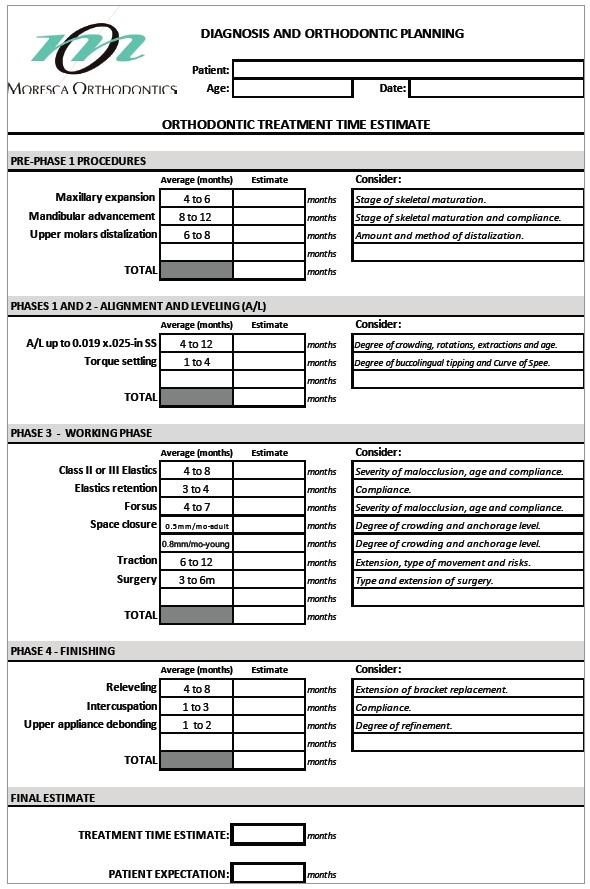
Example of card used to register the time estimate of each orthodontic treatment phase.

» Carry out internal statistics to have an idea about the mean time required for the usual procedures.
» Based on the literature and the aforementioned statistical outcomes, register the time estimate of each treatment phase according to methods and appliances used (Fig 1).

### Orthodontic appliance personalized placement

Despite being of paramount importance, appliance placement does not often receive enough attention. Should it be automated, it might worsen the initial malocclusion, thus hindering correction and unnecessarily adding extra time to treatment.

Precise and individual orthodontic appliance placement might favor correction since treatment onset, thereby enhancing the finishing phase. The estimate is that for each bracket rebonding with a view to increase tooth positioning, treatment time increases 0.3 per month.^16^

#### Suggestion 

» Personalize brackets and tubes placement according to the anatomical shape of the tooth, characteristics of malocclusion and treatment goals.

### Enhancing treatment initial phases

Orthodontic treatment initial phases are those possibly producing the quickest changes. Precious time is wasted when the potential of movement during alignment and leveling is not fully exploited.

On the other hand, many problems are introduced during those phases as a result of uncontrolled tooth movement produced by improper mechanics.

#### Suggestions

» Start tooth movement of both upper and lower arches as soon as possible.
» Fully exploit the potential of movement during alignment and leveling. Precisely plan the desired movements as well as the necessary anchorage.
» Do not postpone procedures planned for this phase, such as interproximal stripping.

### Estimating intervals between appointments

Conventionally, the interval between appointments ranges from three to four weeks. Presently, there is a tendency towards rescheduling appointments with longer intervals in between: from five to six weeks. 

As aforementioned, shorter intervals between appointments can provide better treatment control.

#### Suggestion

» Estimate the interval between appointments according to each treatment phase, the evolution of the case, and patient’s need.

### Keeping focus and organization

Occasionally, the orthodontist may lose focus in terms of time spent during treatment as well as of its goals. Another issue potentially affecting treatment quality is lack of time and attention during appointments.

Such disorganized scenario results in longer treatment with unsatisfactory outcomes. 

#### Suggestions

» Perform thorough examination at each appointment.
» Review treatment planning frequently, to correct occasional diversion.
» Reassess treatment goals at each phase.

### Motivating patients

Keeping patient’s motivation is key to treatment success and to raising patient’s satisfaction. A motivated patient is highly compliant with appointments, hygiene and use of accessory devices. Those factors are critical in controlling orthodontic treatment time.

#### Suggestions

» Often motivate patients with regard to the evolution of treatment.
» Build a relationship with patients, thus raising reliability and treatment satisfaction.

### Avoiding over-refinement

The level of quality required at treatment finishing greatly varies among orthodontists. On the other hand, some occlusal details do not affect function or esthetics and pass unnoticed by patients. A balance among treatment time initially suggested, quality of occlusal refinement and patient’s satisfaction is necessary. 

#### Suggestions

» Start and perform treatment focusing on final outcomes, while trying to foresee potential finishing adjustments.
» Establish high but rather realistic finishing criteria, according to the complexity of the case, time estimate and patient’s demands.
» Share with patients the decision to have appliances debonded.

## FINAL CONSIDERATIONS

Immediacy typical of present times has challenged orthodontists to achieve better outcomes within shorter time. 

Shortening treatment time is a benefit to orthodontists and patients. Therefore, procedures, techniques and appliances aiming at shortening treatment time are valid, provided that they present enough evidence to prove their effectiveness and safety.

Based on current evidence, the most significant factors responsible for determining orthodontic treatment time are orthodontist and patient. Thus, the most highly recommended measures taken to achieve such control are relative to case diagnosis and planning, effective clinical practice and patient compliance.

Treatment-related decisions must be shared with patients, but the orthodontist should not focus on their demands only. Giving priority to esthetics and alignment to provide shorter treatment at all costs and have a higher number of patients is rash. The real challenge faced by current Orthodontics is to balance orthodontic treatment time and quality of outcomes, while trying to achieve, as much as possible, the best esthetic, occlusal and functional goals within reasonable time, according to each case.

With regard to studies found in the literature, it is important to consider that most of them present results based on university research probably carried out with more complex cases treated by training students. For this reason, it is key that each orthodontist takes the literature as reference but also acknowledges his/her own expectation of treatment time for different types of malocclusions.

It is also important to consider that time is a critical factor in orthodontic treatment, as it is necessary for full achievement of angulation and torque, tissue regeneration, stability of outcomes, etc. Too short treatment time may result in incomplete and unstable corrections, with the latter being more susceptible to relapses and consequently requiring longer retention time.

Despite all the effort made by orthodontists and patients, not all variables determining treatment time have been completely enlightened. Therefore, absolute control of orthodontic treatment time is impossible. Potential variations must always be shared with patients.

## CLINICAL CASE 1 (Figs 2 to 4) 

**Figure 2:**
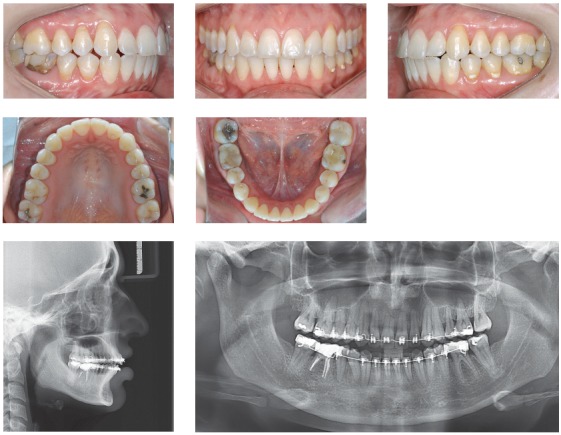
Initial records: intraoral photographs, lateral cephalogram and panoramic radiograph.

**Figure 3:**
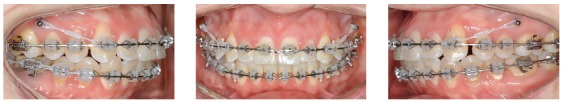
Intraoral photographs showing anterior teeth retraction after molar distalization.

**Figure 4:**
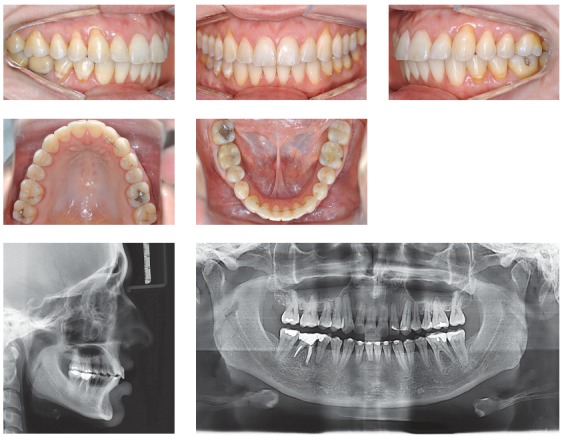
Final records: intraoral photographs, lateral cephalogram and panoramic radiograph.

##  

Female 31-year and 6-month-old patient, presented with canines in 2-mm Class II relationship, upper and lower midlines deviation to the right (2 mm and 3 mm, respectively), and 2.5-mm overjet. Generalized root resorption and vertical bone loss were also found. Cephalometric examination revealed maxillary and mandibular incisors were buccaly tipped. The patient was highly discontented, demotivated and suspicious due to iatrogenesis and two unsuccessful previous orthodontic treatments that together lasted six years.

Treatment planning included minimal tooth movement by means of effective mechanics with mild and controlled forces. Maxillary molars underwent distalization with the aid of miniscrews, to adjust canines in Class I relationship and minimize overjet. Interproximal stripping was carried out on maxillary and mandibular premolars on the left side with a view to enhancing midline adjustment.

The greatest challenge was to balance treatment time between the need for reactivation with longer intervals in between with a view to controlling root resorption and patient’s expectation of having her case solved quickly. There was an attempt to raise patient’s motivation and reliability as the case improved. Treatment lasted 20 months.

## CLINICAL CASE 2 (Figs 5 to 7)

**Figure 5:**
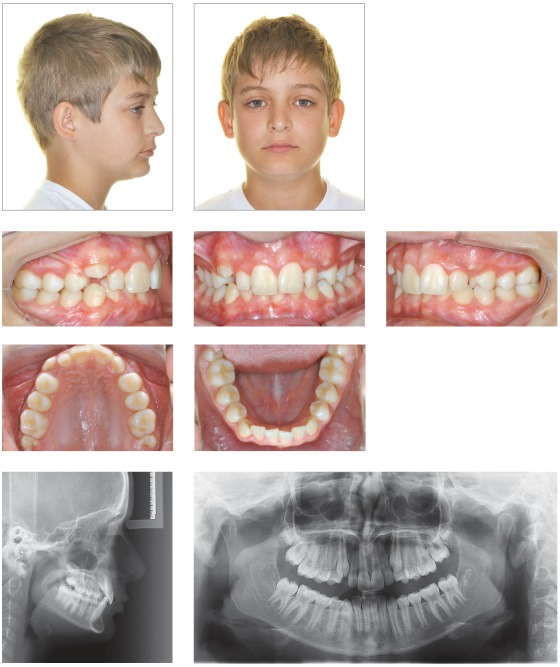
Initial records: extra- and intraoral photographs, lateral cephalogram and panoramic radiograph.

**Figure 6:**
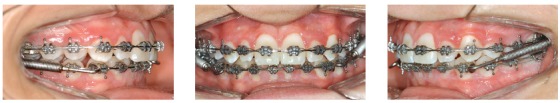
Intraoral photographs showing Class II-correction appliance (Forsus, 3M Unitek).

**Figure 7:**
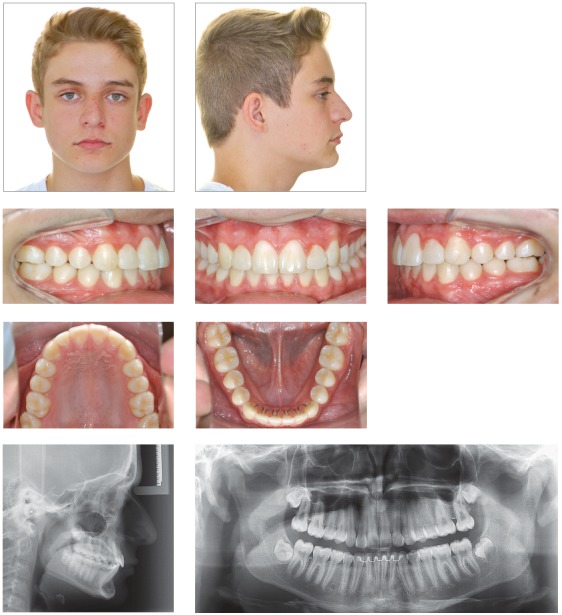
Final records: extra- and intraoral photographs, lateral cephalogram and panoramic radiograph.

Male 11-year and 10-month-old patient, presented with horizontal facial pattern and straight profile, bilateral Class II malocclusion with maxillary incisors lingually tipped towards the left. This resulted in lack of space for maxillary canines, especially on the left side. Shortened mesiodistal width of lateral incisors, especially on the right side. Mandibular incisors were also lingually tipped and extruded, with mild crowding. The patient also presented with deep bite and deep lower curve of Spee. 

Treatment planning included opening spaces for canines and correcting upper midline deviation with proclination of maxillary incisors. Class II was corrected with a Class II-correction intraoral fixed appliance. Esthetic restorations were performed on maxillary lateral incisors in order to have mesiodistal width adjusted.

The greatest concern was controlling orthodontic treatment time, with a view to minimizing issues resulting from low hygiene compliance. To this end, low-friction mechanics was used to enhance treatment initial phases, in addition to a Class II-correction fixed appliance. Some occlusal details could have been improved; however, the aim was not to extend treatment time and have appliances debonded after 18 months. This is because esthetic and functional outcomes were considered satisfactory.

## CLINICAL CASE 3 (Figs 8 to 10)

**Figure 8:**
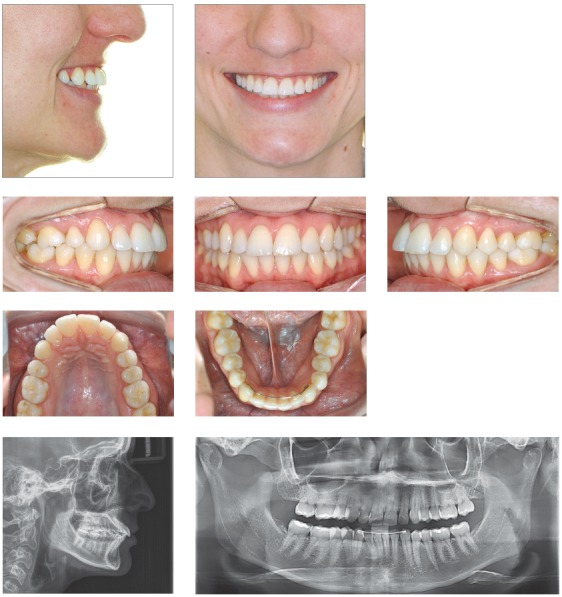
Initial records: extra- and intraoral photographs, lateral cephalogram and panoramic radiograph.

**Figure 9:**
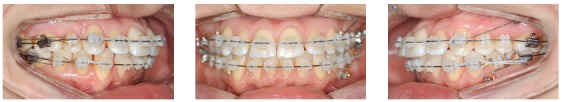
Intraoral photographs showing incisors retraction.

**Figure 10:**
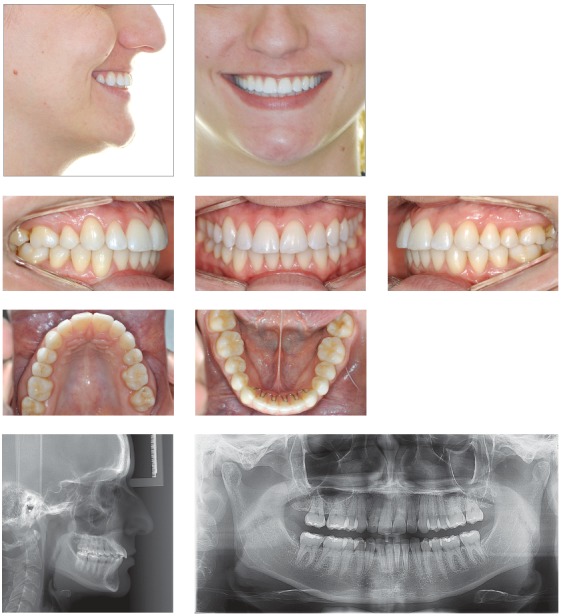
Final records: extra- and intraoral photographs, lateral cephalogram and panoramic radiograph.

Female 26-year-old patient, whose chief complaint was maxillary incisors protrusion and upper midline deviation to the right due to unilateral right maxillary premolar extraction recommended by her previous orthodontist. The patient present with Class II molar relationship on the left side and Class I molar relationship on the right side. 

The patient was willing to use orthodontic appliances for nine months only, due to personal reasons. Once treatment planning had been approved and other possibilities had been presented, final planning included upper midline deviation correction and maxillary incisors protrusion improvement.

Interproximal stripping was performed on mandibular premolars to allow for some mandibular incisors retraction. It was also performed on maxillary premolars on the left side, to correct upper midline deviation. Miniscrews were used on the left side to allow for more effective movement. Treatment time remained within the initial 9-month expectation. To have buccolingual incisors tipping stabilized, esthetic aligners were used as retainers.

## CONCLUSIONS

Treatment time varies according to the type of malocclusion and treatment options. Orthodontist’s influence, patient’s characteristics and compliance are all decisive in determining treatment time, while the effects provided by orthodontic appliances and methods used to speed tooth movement up seem little effective. Simple clinical strategies can contribute to control and shorten orthodontic treatment time.
